# Multicenter Retrospective Study of Invasive Fusariosis in Intensive Care Units, France

**DOI:** 10.3201/eid3002.231221

**Published:** 2024-02

**Authors:** Jordane Demonchy, Lucie Biard, Raphaël Clere-Jehl, Florent Wallet, Djamel Mokart, Anne-Sophie Moreau, Laurent Argaud, Camille Verlhac, Frédéric Pène, Alexandre Lautrette, Naïke Bige, Audrey de Jong, Emmanuel Canet, Jean-Pierre Quenot, Nahéma Issa, Yoann Zerbib, Inès Bouard, Muriel Picard, Lara Zafrani

**Affiliations:** Centre Hospitalier Universitaire de Lille, Lille, France (J. Demonchy, A.-S. Moreau);; Institut national de la santé et de la recherche médicale (INSERM), University of Paris Cité, Paris, France (J. Demonchy, L. Zafrani);; Hôpital Saint-Louis, Paris (L. Biard, I. Bouard, L. Zafrani);; Centre Hospitalier Universitaire de Strasbourg, Strasbourg, France (R. Clere-Jehl);; Centre Hospitalier Universitaire de Lyon, Lyon, France (F. Wallet, L. Argaud);; Institut Paoli-Calmettes, Marseille, France (D. Mokart);; Centre Hospitalier Universitaire de Clermont-Ferrand, Clermont-Ferrand, France (C. Verlhac, A. Lautrette);; Hôpital Cochin, Paris (F. Pène); Hôpital Saint-Antoine, Paris (N. Bige);; Montpellier University, INSERM, and St-Eloi Hospital, Montpellier, France (A. de Jong); Centre Hospitalier Universitaire de Nantes, Nantes, France (E. Canet);; Centre Hospitalier Universitaire de Dijon Bourgogne, Dijon, France (J.-P. Quenot);; Centre Hospitalier Universitaire de Bordeaux, Bordeaux, France (N. Issa);; Centre Hospitalier Universitaire de Amiens-Picardie, Amiens, France (Y. Zerbib);; Centre Hospitalier Universitaire de Toulouse, Toulouse, France (M. Picard)

**Keywords:** Fusarium, fungi, antimicrobial resistance, invasive fusariosis, intensive care unit, hematologic malignancy, France

## Abstract

Invasive fusariosis can be life-threatening, especially in immunocompromised patients who require intensive care unit (ICU) admission. We conducted a multicenter retrospective study to describe clinical and biologic characteristics, patient outcomes, and factors associated with death and response to antifungal therapy. We identified 55 patients with invasive fusariosis from 16 ICUs in France during 2002­–­­2020. The mortality rate was high (56%). Fusariosis-related pneumonia occurred in 76% of patients, often leading to acute respiratory failure. Factors associated with death included elevated sequential organ failure assessment score at ICU admission or history of allogeneic hematopoietic stem cell transplantation or hematologic malignancies. Neither voriconazole treatment nor disseminated fusariosis were strongly associated with response to therapy. Invasive fusariosis can lead to multiorgan failure and is associated with high mortality rates in ICUs. Clinicians should closely monitor ICU patients with a history of hematologic malignancies or stem cell transplantation because of higher risk for death.

## Abstract 2

Elevated SOFA scores at admission or history of allogeneic hematopoietic stem cell transplantation or hematologic malignancies were associated with death.

Invasive fungal infections are common, and severe complications can occur in immunocompromised patients, especially in patients with hematologic malignancies who require intensive care unit (ICU) admission (*1*,*2*). Invasive fusariosis is a mycosis caused by infection with *Fusarium* spp. (*3*). *Fusarium* are ubiquitous filamentous fungi that can cause a range of infections, from localized lesions due to penetrating trauma in healthy persons, to acute invasive or disseminated infection in immunocompromised patients (*3*–*6*). Most frequent clinical manifestations of invasive fusariosis are fever refractory to antimicrobial drugs, pneumonia, metastatic skin lesions of a disseminated infection, and sinusitis (*3*,*4*,*6*).

The European Organization for Research and Treatment of Cancer/Invasive Fungal Infections Cooperative Group and the US National Institute of Allergy and Infectious Diseases Mycoses Study Group (EORTC/MSG) published definitions for proven and probable invasive fusariosis in immunocompromised patients (*7*). Although proven infection requires microscopic analysis or culture of a sterile material, probable infection is based on host factors, clinical features, and mycologic criteria (Appendix Table 1). Despite progress in managing invasive fungal infections in recent decades, including the widespread use of antifungal prophylaxis in immunocompromised patients and improved treatment strategies, invasive fusariosis remains a serious and potentially life-threatening infection. Invasive fusariosis can lead to severe organ failure and has been associated with mortality rates ranging from 40%­ to 70% (*8*–*11*). Even when amphotericin B and voriconazole are the first drugs of choice, sometimes in combination, the best antifungal treatment remains unclear (*12*).

Data focusing on fusariosis rely mainly on case reports (*13*–*15*), studies based on selected populations (*9*–*11*,*16*,*17*), or epidemiologic studies (*18*,*19*). None of those studies focused on critically ill patients with invasive fusariosis. We conducted a multicenter retrospective study to describe the characteristics and outcomes of invasive fusariosis in ICU patients in France and to identify the main risk factors associated with death and response to therapy.

## Methods

### Ethics

This observational study was based on anonymized hospitalization reports and was in strict compliance with the reference methodology MR-004 of France. The study was approved by the data protection authority, Commission Nationale de l’Informatique et des Libertés (registration no. 2220799v0), and received a favorable opinion from the Comité Ethique de la Société de Réanimation de Langue Française institutional review board (approval no. 20–95). The study was conducted in accordance with principles of the Declaration of Helsinki (World Medical Association, https://www.wma.net).

### Study Population

**Figure 1:**
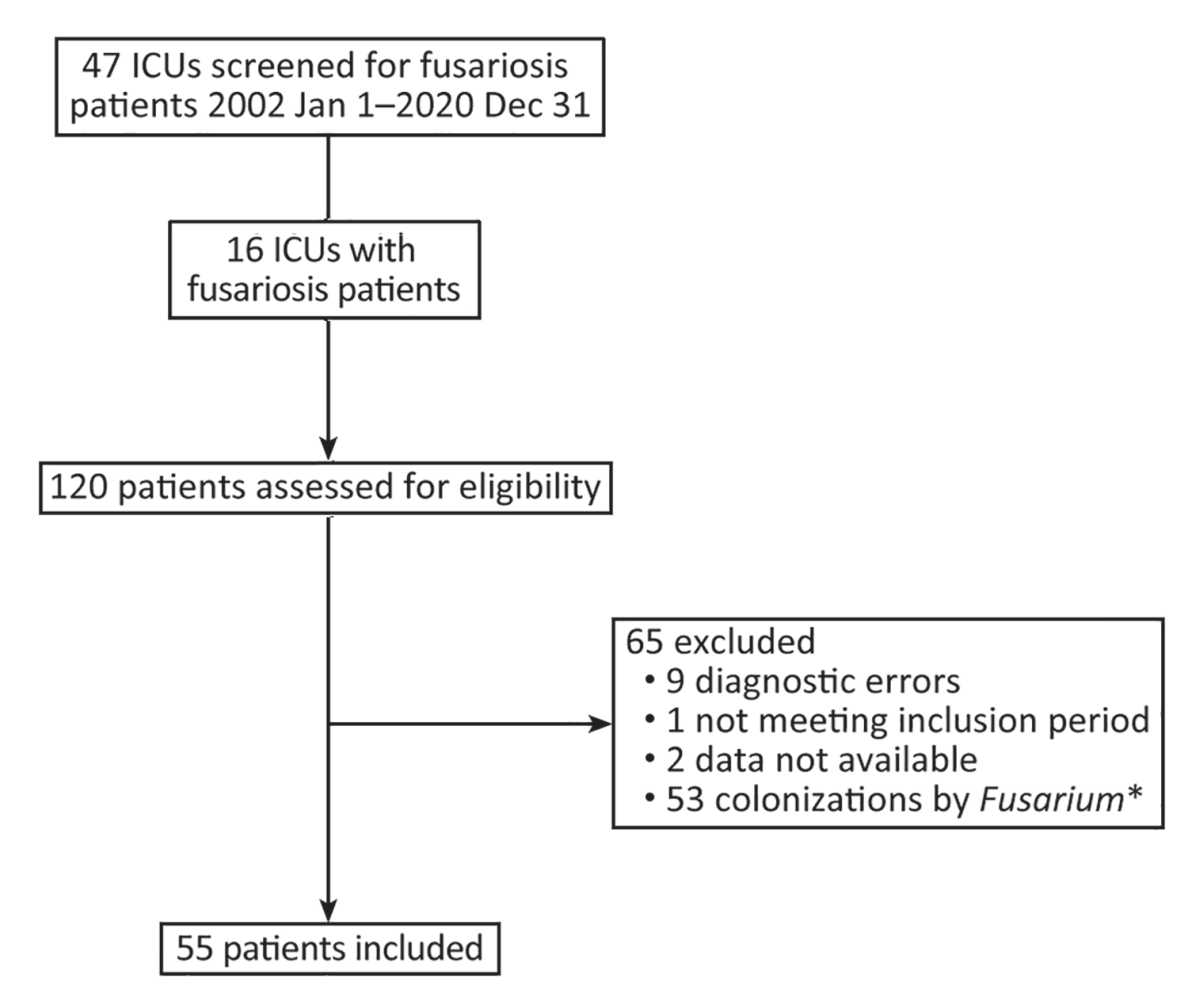
Flowchart for patient inclusion in a multicenter retrospective study of invasive fusariosis in ICUs, France. *Colonization by *Fusarium* defined as patient not meeting the European Organization for Research and Treatment of Cancer/Invasive Fungal Infections Cooperative Group or National Institute of Allergy and Infectious Diseases Mycoses Study Group criteria for proven or probable invasive fungal diseases (*7*). ICU, intensive care unit.

We retrospectively included in the study adult ICU patients with a diagnosis of invasive fusariosis during January 1, 2002–December 31, 2020. We used a modified EORTC/MSG criteria to determine diagnosis of proven or probable invasive fusariosis (*7*) (Appendix Table 1). We identified patients by reviewing medical records, microbiologic databases, or both. To identify patients eligible for our study, we conducted a comprehensive screening of all ICUs and parasitology and mycology departments in France from which *Fusarium* species had been identified. Of 47 screened ICUs, only 16 had patients with a positive *Fusarium* microbiologic documentation during the inclusion period. We assessed a total of 120 patients for eligibility. We excluded 53 patients with *Fusarium* colonization and ultimately included 55 patients in the study (Figure 1).

We collected data from anonymized hospitalization reports, including information on patient age, sex, underlying disease conditions, history of immunodeficiency, clinical and microbiologic characteristics of the *Fusarium* infection, any co-infections, antifungal treatment, need for organ support, and outcomes. For each patient, simplified acute physiology score (SAPS II) and sequential organ failure assessment (SOFA) scores were collected at admission, as previously defined (*20*,*21*). For response to therapy, we defined progression as clinical deterioration or death after antifungal treatment. We considered response complete if clinical improvement occurred, biological samples became sterile, and computed tomography (CT) features cleared. When all the complete response criteria were not met, we considered response to therapy to be partial.

### Outcomes

Our primary objective was to identify factors associated with ICU mortality rates. Our secondary objective was to identify factors associated with response to therapy.

### Statistical Analyses

We reported continuous variables as medians and interquartile ranges (IQRs) and categorical variables as numbers and percentages. We considered response to treatment, death in the ICU, death in the hospital, and death within 1 year as binary endpoints for the main analysis. We compared continuous variables by using Wilcoxon rank-sum test and compared categorical variables by using Fisher exact test. We performed adjusted analyses to evaluate factors associated with treatment response and death by using multivariable logistic regression models. We estimated cumulative incidence of death in the ICU by using standard methods for competing events and considered discharged alive as a competing outcome to ICU death. We performed all tests 2-sided at the 5% significance level. We performed all analyses on the R statistical platform (The R Foundation for Statistical Computing, https://www.r-project.org).

## Results

### Patient Characteristics

We identified 55 patients with invasive fusariosis in 16 ICUs during the inclusion period (Table 1). The median age was 61 (IQR 52­–67) years; 80% (n = 44) were immunocompromised, most (n = 32) of whom had active hematologic malignancies (36%, n = 16) or underwent a recent (<1 year) allogeneic hematopoietic stem cell transplantation (allo-HSCT) (36%, n = 16). Eleven (25%) immunocompromised patients had a medical history of solid organ transplantation. Of 11 (20%) immunocompetent patients, all had invasive fusariosis diagnosed during a prolonged (>2 weeks) ICU hospitalization, including hospitalization for septic shock (n = 7), acute respiratory distress syndrome (n = 2), and multiple traumatic injuries (n = 2). Only 24% (n = 13) of patients had an antifungal prophylaxis at ICU admission. Patients admitted to the ICU had severe illness as indicated by elevated median SAPS II and SOFA scores.

**Table 1:** Clinical and biologic characteristics among 55 patients in a multicenter retrospective study of invasive fusariosis in intensive care units, France*

Characteristics	Value
Median age, y (IQR)	61 (52–67)
Sex	
M	39 (71)
F	26 (29)
Immunocompromise type	44 (80)
Hematologic malignancy	16 (36)
Allo-HSCT	16 (36)
Solid organ transplant	11 (20)
Kidney transplant	2 (18)
Liver transplant	4 (36)
Kidney-liver transplant	1 (9)
Lung transplant	3 (27)
Heart transplant	1 (9)
Rheumatoid arthritis with corticosteroids	1 (2)
Diabetes mellitus	10 (18)
Immunosuppressive agents	
Corticosteroids >3 weeks	12 (22)
Other immunosuppressive therapy	22 (40)
Chemotherapy <3 mo	21 (38)
Biologic data	
Neutropenia, neutrophil <0.5 G/L	22 (40)
Lymphopenia, lymphocytes <1 G/L	47 (85)
Hypoalbuminemia, albumin <35 g/L	48 (87)
Antifungal prophylaxis	13 (24)
Posaconazole	9 (16)
Voriconazole	2 (4)
Fluconazole	2 (4)
Performance status at admission	
>2	30 (55)
<2	25 (45)
Median prognostic scores at admission (IQR)	
SAPS II	54 (40–65)
SOFA at admission	9 (6–13)

*Values are no. (%) except as indicated. Allo-HSCT, allogeneic hematopoietic stem cell transplantation; IQR, interquartile range; SAPS II, simplified acute physiology score II; SOFA, sequential organ failure assessment.

During ICU stays, acute respiratory failure was the main organ failure in patients with invasive fusariosis; 80% (n = 44) of patients required invasive mechanical ventilation (Table 2). Furthermore, acute kidney injury was observed in 73% (n = 40) of patients, among whom 29 (72.5%) required renal-replacement therapy, such as continuous venovenous hemofiltration and hemodialysis. The incidence of acute kidney injury was notably higher (100%) for the 11 patients with solid organ transplant than for the patients with hematologic malignancies (44%, n = 7), allo-HSCT (69%, n = 11), and other patients (92%, n = 11) (p = 0.003) (Appendix Table 2).

**Table 2:** Organ failure and outcomes among 55 patients in a multicenter retrospective study of invasive fusariosis in intensive care units, France*

Characteristics	Value
Invasive mechanical ventilation†	44 (80)
Median days (IQR)	16 (9–39)
Noninvasive ventilation	18 (33)
High flow nasal oxygen therapy	11 (20)
Prone position	5 (9)
Neuromuscular blockers	13 (24)
Nasal oxygenotherapy	49 (89)
Vasopressors	38 (69)
Acute kidney injury	40 (73)
Renal-replacement therapy	29 (53)
Acute liver failure	18 (33)
Median length of stay, d (IQR)	17 (6–37)
Death in ICU	31 (56)

*Values are no. (%) except as indicated. IQR, interquartile range. With orotracheal intubation cannula or tracheotomy.

Patients experienced prolonged ICU hospitalizations; median length of stay was 17 (IQR 6­–37) days, and the mortality rate was high (56%, n = 31). Of 31 ICU patients who died, 18 (58.1%) deaths were considered directly related to invasive fusariosis and 13 (41.9%) deaths were not considered directly related to invasive fusariosis. Of those 13 deaths, causes were multivisceral organ failures related to secondary infections (n = 6), severe graft versus host disease (n = 1), progression of the underlying malignancy (n = 2), hemorrhagic shock (n = 1), or withdrawal of life-sustaining treatment (n = 3). Among ICU survivors, 1 (4%) patient died in the hospital and 3 (4%) patients died within 1 year of diagnosis.

### Invasive Fusariosis in ICUs

**Figure 2:**
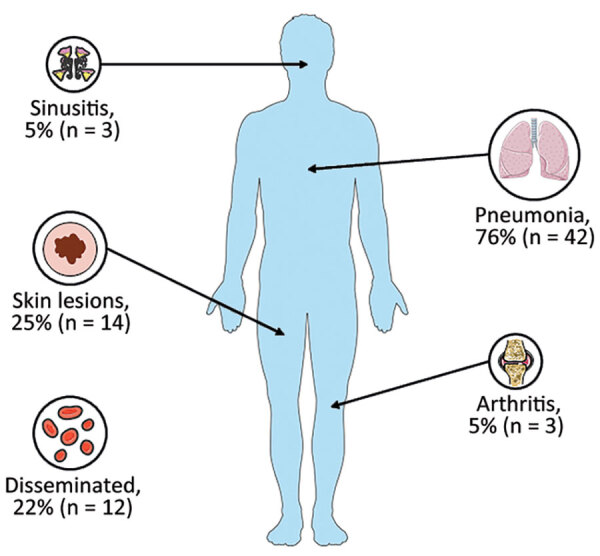
Main clinical manifestations among patients in a multicenter retrospective study of invasive fusariosis in intensive care units, France. The figure was partly generated using Servier Medical Art (https://smart.servier.com), licensed under a Creative Commons Attribution 3.0 unported license.

Using EORTC/MSG criteria, we classified a total of 32 (58%) cases as probable invasive fusariosis and 23 cases (42%) as proven invasive fusariosis (Table 3). Among invasive fusariosis patients, 53% of diagnoses were established after admission to the ICU; median time from ICU admission to invasive fusariosis diagnosis was 9 (IQR 1–16) days. Mycologic diagnosis was achieved through culture of various biologic samples and was guided by the patients’ clinical signs and symptoms. Blood cultures (22%, n = 12) were used for cases of fever and disseminated invasive fusariosis, biopsies (29%, n = 10) were taken from skin lesions, sputum (29%, n = 16) and bronchoalveolar lavage fluid (22%, n = 12) were collected for pneumonia cases, sinus aspirate samples (5%, n = 3) were obtained for sinusitis, joint fluid (5%, n = 3) was examined for arthritis, and pancreatic fluid (2%, n = 1) collections were analyzed for suspected infection (Figure 2). Pathologic examination of skin or sinus biopsies revealed *Fusarium* associated with tissue damage in 10 (18%) patients. 

**Table 3:** Characteristics of *Fusarium* infections and co-infections in among 55 patients in a multicenter retrospective study of invasive fusariosis in intensive care units, France

Infections and co-infections	No. (%) patients
Fusariosis diagnosis	
Probable	32 (58)	
Proven	23 (42)
*Fusarium* species	
*Fusarium* spp.	38 (69)	
* F. oxysporum*	5 (9)	
* F. proliferatum*	4 (7)	
* F. solani*	3 (5)	
* F. fujikuroi*	2 (4)	
* F. dimerum*	1 (2)	
* F. monoliforme*	1 (2)	
* F. keratoplasticum*	1 (2)
Mycologic diagnosis	
Mycologic culture of biologic samples*	55 (100)
Pathologic examination of biopsies†	10 (18)
Positive serum galactomannan	15 (27)
Time of diagnosis from intensive care admission	
Before admission	12 (22)	
Day of admission	12 (22)	
After admission	29 (53)
Clinical manifestation	
Disseminated infection	12 (22)
Skin lesions	14 (25)
Pneumonia	42 (76)
Sinusitis	3 (5)	
Arthritis	3 (5)	
Infection of pancreatic fluid collections	1 (2)
Thoracic computed tomography patterns of pneumonia, n = 37	
Pulmonary consolidations	16 (43)
Nodules and micronodules	12 (32)
Excavated pulmonary lesions	3 (8)
Ground glass opacities	9 (24)
Pleural effusion	6 (16)
Missing data	5 (14)
Co-infections	
Bacterial	32 (58)	
Viral	19 (35)	
Fungal	27 (49)
Antifungal treatment‡	
Monotherapy	46 (84)	
Voriconazole	23 (42)	
Amphotericin B	21 (38)	
Isavuconazole	1 (2)	
Terbinafine	1 (2)	
Combination therapy		
Voriconazole + amphotericin B	11 (20)	
Isavuconazole + micafungin	1 (2)	
None	4 (7)	
Missing data	1 (2)
Granulocyte colony-stimulating factor	12 (22)
Surgical debridement of localized infection	7 (13)
Response to therapy	
Progression	28 (51)	
Partial or complete	14 (25)	
Missing data	13 (24)

*Biologic samples included blood cultures, skin biopsies, sputum, bronchoalveolar lavage fluid, sinus aspirate samples, joint fluid, and pancreatic fluid collections. †Pathologic examination of skin or sinus biopsies in which *Fusarium* are seen and accompanied by tissue damage. ‡Some patients first received a monotherapy and then a combination of 2 drugs because of refractory disease.

Results of serum galactomannan test were available for 50 (90.9%) patients and 15 (30%) of them had a positive serum galactomannan test on the day of invasive fusariosis diagnosis. Among those patients, 4 also had concomitant aspergillosis diagnosed. Other observed co-infections included bacterial co-infection in 58% (n = 32), viral co-infection in 35% (n = 19), and fungal co-infection in 34% (n = 19) of invasive fusariosis patients (Appendix Table 3). 

**Figure 3:**
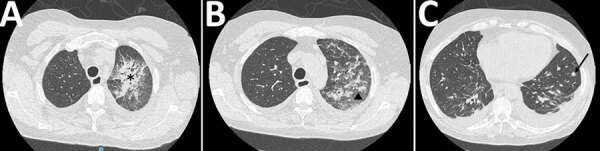
Thoracic computed tomography scans from patients included in a multicenter retrospective study of invasive fusariosis in intensive care units, France, showing findings of fusariosis-related pneumonia. A) Unilateral consolidations (asterisk); B) ground glass opacities (triangle); C) 7-mm nodule (arrow) and bilateral pleural effusion.

Pneumonia was the most prevalent clinical manifestation, accounting for 76% (n = 42) of the cases. Consistent with the EORTC/MSG criteria, the diagnosis of fungal lung disease primarily relied on thoracic CT. Among the patients, fusariosis-related pneumonia exhibited a wide range of thoracic CT patterns (Figure 3): 43% (n = 16) had pulmonary consolidations, 32% (n = 12) had nodules and micronodules, 24% (n = 9) had ground glass opacities, 16% (n = 6) had pleural effusion, and 8% (n = 3) had excavated pulmonary lesions. Moreover, the incidence of disseminated invasive fusariosis was notably higher (44%, n = 7) in patients with hematologic malignancies than in patients who had allo-HSCT (25%, n = 4) or solid organ transplants (9%, n = 1) (p = 0.03) (Appendix Table 2).

Two thirds of invasive fusariosis patients received antifungal monotherapy. The 2 primary drugs used were voriconazole (62%, n = 34) and amphotericin B (60%, n = 33). Four (7%) patients died before invasive fusariosis diagnosis and did not receive treatment. Granulocyte colony-stimulating factor was administered to 55% (n = 12) of the neutropenic patients, and surgical debridement of localized lesions was performed in 13% (n = 7) of patients. Half of the patients experienced disease progression despite receiving adequate therapy.

### Factors Associated with Response to Therapy

Factors associated with invasive fusariosis progression under therapy in all 55 patients by univariate analysis were history of recent (<1 year) and past (>1 year) allo-HSCT (p = 0.049), corticosteroid therapy for >3 weeks (p = 0.019), a higher SOFA score at admission (p = 0.002), performance status >2 at admission (p = 0.022), and pulmonary consolidations on thoracic CT for fusariosis-related pneumonia (p = 0.001) (Appendix Table 4). Conversely, nodules and micronodules on thoracic CT were significantly associated with partial and complete response (p = 0.001). By multivariate analysis, none of the following were significantly associated with response to therapy: voriconazole treatment (odds ratio [OR] 3.55, 95% CI 0.72–17.6; p = 0.12), history of allo-HSCT (OR 0.21, 95% CI 0.036–1.24; p = 0.086), and disseminated fusariosis (OR 0.15, 95% CI 0.015–1.42; p = 0.098).

### Factors Associated with Death

By univariate analysis, signs and symptoms significantly associated with death in the ICU included history of hematologic malignancies and allo-HSCT (p = 0.017), immunosuppressive therapy other than corticosteroids (p = 0.036), elevated SAPS II (p = 0.007) or SOFA (p = 0.001) score at admission, and neutropenia (neutrophils <0.5 G/L) (p = 0.05) (Appendix Table 5). Among patients with organ failure, only vasopressors were associated with death (p = 0.006). Conversely, surgical debridement of localized lesion was associated with ICU survival (p = 0.014).

**Figure 4:**
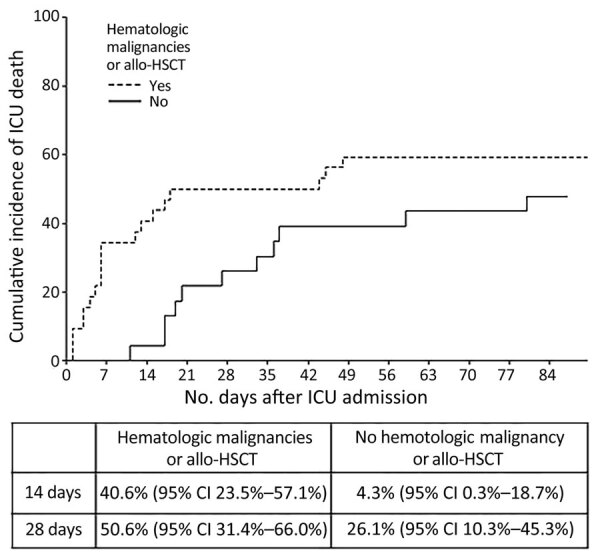
Cumulative incidence of death among patients with and without history of hematologic malignancies and allo-HSCT in a multicenter retrospective study of invasive fusariosis in ICUs, France. Calculations used Fisher exact test (p = 0.017). Chart shows 14-day and 28-day death rates. Allo-HSCT, allogeneic hematopoietic stem cell transplant; ICU, intensive care unit.

By multivariate analysis, the factors associated with death in the ICU were higher SOFA score (OR 1.51, 95% CI 1.15–1.98; p = 0.003) and history of hematologic malignancy or allo-HSCT (OR 8.28, 95% CI 1.26–54.2; p = 0.027). Cumulative incidence of ICU death showed a 50% (95% CI 31.4%–66%) ICU mortality rate at 28 days for patients with hematologic malignancies or allo-HSCT compared with 26.1% (95% CI 10.3%–45.3%) for patients without hematologic malignancy and allo-HSCT (Figure 4). Multivariate analyses on factors associated with death in the hospital and within 1 year of admission were similar to the results of the analyses for factors associated with death in the ICU. Indeed, higher SOFA score was associated with death in the hospital (OR 1.50, 95% CI 1.14–1.97; p = 0.004), death within 1 year of admission (OR 1.66 95% CI 1.16–2.36; p = 0.005), history of hematologic malignancy (OR 7.87, 95% CI 1.18–52.6; p = 0.033 ), or allo-HSCT (OR 15.3, 95% CI 1.60–145.7; p = 0.018).

## Discussion

We conducted a large retrospective study to describe the clinical characteristics and outcomes of patients with invasive fusariosis admitted to ICUs in France. We found that invasive fusariosis can be life-threatening; often is associated with bacterial, viral, and fungal co-infections; and occurs mainly in immunocompromised patients or patients enduring extended ICU stays. Pneumonia is the prevailing clinical manifestation in ICU patients. Despite ICU hospitalization, organ support, and adequate treatment, the fusariosis mortality rate remains high. SOFA score and a history of allo-HSCT or hematologic malignancies, or both, are significantly associated with death in the ICU.

The available medical literature on invasive fusariosis remains scarce, and a paucity of studies specifically focused on invasive fusariosis in the ICU setting. The few studies dedicated to invasive fungal infections in ICU patients included <5 patients with invasive fusariosis (*2*,*22*). Our study provides a comprehensive description of clinical, biologic, and microbiologic characteristics of critically ill invasive fusariosis patients. 

Previous studies indicate that most invasive fusariosis patients have immunocompromising conditions, including hematologic malignancies, recent allo-HSCT, or solid organ transplantation (*9*,*11*,*17*,*19*,*23*). However, we found that one fifth of patients with invasive fusariosis in the ICU are considered immunocompetent at ICU admission but experienced a prolonged ICU hospitalization, mainly because of septic shock. That finding supports the concept of sepsis-induced immunosuppression, wherein an imbalanced inflammatory state contributes to immunoparalysis and increases the risk for nosocomial infections (*24*). Therefore, physicians should investigate the possibility of invasive fusariosis in patients with prolonged ICU hospitalization, especially in cases of a secondary sepsis unresponsive to antimicrobial agents and clinical manifestations consistent with invasive fusariosis.

The clinical manifestations we observed in our cohort align with those from previous reports (*3*,*4*,*6*). However, a notable finding in our study was identification of *Fusarium* in mycologic culture (semiquantitative results showing numerous *Fusarium* colonies) from pancreatic fluid collected in a case of suspected infection from a site that has not been previously described. That novel observation highlights the importance of considering *Fusarium* as a potential pathogen in unusual infection sites and expands our understanding of clinical manifestations of fusariosis. In addition, our study revealed a lower prevalence of disseminated infection, affecting only one fifth of patients, in contrast to reports from previous publications focusing on non-ICU patients (*8*,*11*,*17*). However, patients with hematologic malignancies in our study exhibited much higher rates of disseminated infections. That finding aligns with a hypothesis proposed by others that suggests the larger proportion of neutropenia in fusariosis patients might contribute to the increased susceptibility to disseminated infection (*17*).

We noted a marked predominant prevalence of pneumonia (76%) among our study population. That finding highlights that fusariosis-related pneumonia can lead to acute respiratory failure, often necessitating invasive mechanical ventilation. Thoracic CT patterns of fusariosis-related pneumonia included pulmonary consolidations, micronodules and nodules, and ground-glass opacities. Excavations and pleural effusions have also been observed, but proportions of those CT patterns vary across different publications, mainly due to the small number of patients included (*25*–*27*). In addition, the timing of imaging and presence of neutropenia or co-infections might influence those patterns. Many patients in our study had co-infections; thus, we cannot attribute their CT patterns solely to invasive fusariosis.

All patients in our study who had solid organ transplants also had acute kidney injury. That difference varied from previous reports and could be explained mainly by the presence of calcineurin inhibitor, well known for its nephrotoxicity (*28*). In addition, one third of solid organ transplant patients in our study had undergone a kidney transplant, which might have contributed to the increased susceptibility to acute kidney injury in this subgroup.

Despite identifying various factors associated with treatment response in the univariate analysis, the multivariate analysis in our study did not reveal any independent risk factors. However, the small number of patients included in our study might have limited the statistical power of the analysis. Moreover, we considered all non-ICU survivors to be nonresponders and 4 patients died before receiving treatment, findings others should consider when interpreting our results but that further emphasize the need for larger studies among more extensive patient populations to better elucidate the independent risk factors associated with treatment response in invasive fusariosis. 

The optimal antifungal treatment for invasive fusariosis remains uncertain (*29*). The heterogeneity of treatments administered to patients across different centers in our study further complicates the interpretation of our results. However, our analysis was underpowered to detect a favorable response with voriconazole. Conversely, a previous study reported a 90-day survival rate of 60% with voriconazole monotherapy (*8*), surpassing the outcomes associated with liposomal or deoxycholate amphotericin B. Another study demonstrated an overall response rate of 47% with voriconazole (*10*). Nevertheless, because the current literature primarily consists of case reports and small retrospective studies, determining the optimal antifungal regimen for such patients remains challenging.

The mortality rate observed for ICU patients with invasive fusariosis in our study was notably high, reaching 56%. That finding is consistent with previous studies reporting mortality rates ranging from 40% to 70% in patients with invasive fusariosis, although those studies did not specifically focus on ICU patients (*8*–*11*). In univariate analysis, the observed association between surgical debridement and survival could be attributed to the fact that patients were well enough, and possibly had less severe and fewer disseminated infections, to undergo debridement. By multivariate analysis, we identified history of hematologic disease, including active hematologic malignancy or recent allo-HSCT, as an independent risk factor for death. Patients with hematologic malignancies and those who have undergone allo-HSCT are more likely to experience neutropenia. Persistent neutropenia has been identified as a factor associated with increased mortality rates among invasive fusariosis patients in several previous studies (*8*,*16*,*17*,*30*). 

One limitation in our study is the lack of assessment of persistent neutropenia during hospitalization because of missing data on hospitalization reports; those missing data prevented a comprehensive analysis of the effects of persistent neutropenia on patient outcomes in our study population. Also, because we did not have access to the total number of immunocompromised patients admitted to ICUs during the entire study period, we were unable to estimate the prevalence of invasive fusariosis in this population. Finally, the variability in ICU admitting policies across different centers might have influenced our study results. Some patients with invasive fusariosis and underlying conditions or poor prognosis related to hematologic malignancies might have been denied ICU admission. That potential selection bias could affect the generalizability of our findings and should be considered when interpreting the results. 

In conclusion, invasive fusariosis is a severe condition that can lead to multiorgan failure and is associated with high mortality rates in the ICU. Clinicians should consider invasive fusariosis as a potential diagnosis in immunocompromised patients who have pneumonia or persistent fever unresponsive to antimicrobial agents. Treatment for invasive fusariosis includes antifungal therapy, rapid reversal of neutropenia, and surgical debridement for localized lesions. Further research is warranted to optimize diagnostic strategies and treatment approaches for this challenging and life-threatening infection. However, clinicians should closely monitor ICU patients with a history of hematologic malignancies or allo-HSCT because of significantly higher invasive fusariosis ICU mortality rates among those patients. 

Appendix Additional information on a multicenter retrospective study of invasive fusariosis in intensive care units, France.
