# Supplementary material for: Multicenter Retrospective Study of Invasive Fusariosis in Intensive Care Units, France
Source: Emerg Infect Dis. 2024 Feb;30(2):215–24. doi: 10.3201/eid3002.231221 (PMC10826781; doi:10.3201/eid3002.231221)
Supplement: Appendix — Additional information on a multicenter retrospective study of invasive fusariosis in intensive care units, France. [file 23-1221-Techapp-s1.pdf]

Article DOI: https://doi.org/10.3201/eid3002.231221

EID cannot ensure accessibility for supplementary materials supplied by authors. Readers who have difficulty accessing supplementary content should contact the authors for assistance.

Multicenter Retrospective Study of Invasive Fusariosis in Intensive Care Units, France

Appendix

Appendix Table 1. EORTC/MSG criteria for proven and probable invasive fusariosis disease*

Criteria for proven and probable fusariosis
Proven invasive fusariosis
Microscopic analysis† of sterile material obtained by needle aspiration or biopsy
Culture of sterile material‡ (excluding bronchoalveolar lavage fluid, cranial sinus specimen and urine)
Blood culture positive for *Fusarium* species
Probable invasive fusariosis
Host factors
Neutropenia (neutrophils <0.5 g/L) for >10 d
Allogeneic hematopoietic stem cell transplant
Prolonged use of corticosteroids >3 weeks
Treatment with other T cell immunosuppressants§ during the past 3 mo
Inherited severe immunodeficiency
Clinical criteria
Lower respiratory tract fungal disease with signs on computed tomography scan
Tracheobronchitis seen on bronchoscopic analysis
Sinonasal infection with clinical and imaging signs
Mycologic criteria
Direct test (cytologic, microscopic analysis, or culture) of sputum, bronchial brush, bronchoalveolar lavage fluid, sinus aspirate samples positive for *Fusarium* species

*Adapted from De Pauw B, Walsh TJ, Donnelly JP, Stevens DA, Edwards JE, Calandra T, et al.; European Organization for Research and Treatment of Cancer/Invasive Fungal Infections Cooperative Group; National Institute of Allergy and Infectious Diseases Mycoses Study Group (EORTC/MSG) Consensus Group. Revised definitions of invasive fungal disease from the European Organization for Research and Treatment of Cancer/Invasive Fungal Infections Cooperative Group and the National Institute of Allergy and Infectious Diseases Mycoses Study Group (EORTC/MSG) Consensus Group. Clin Infect Dis. 2008;46:1813–21. https://doi.org/10.1086/588660.
†Histopathologic, cytopathologic, or direct microscopic examination.
‡Specimen obtained by a sterile procedure from a normally sterile and clinically or radiologically abnormal site consistent with an infectious disease process Probable invasive fusariosis requires the presence of host factor, a clinical and mycological criterion.
§Such as cyclosporine, TNFα blockers, specific monoclonal antibodies, or nucleoside analogs.

Appendix Table 2. Univariate analysis of clinical, microbiological, radiological features and organ failures of invasive fusariosis patients according to the underlying immunodeficiency

Features	Hematologic malignancies, n = 16	Allo-HSCT, n = 16	Solid organ transplant, n = 11	Other, n = 12	p value
Clinical presentation					
Disseminated	7 (44)	4 (25)	1 (9)	0	**0.03**
Skin lesions	4 (25)	5 (31)	2 (18)	3 (25)	0.97
Pneumonia	13 (81)	12 (75)	10 (91)	7 (58)	0.31
Other	2 (13)	2 (13)	0	3 (25)	0.39
Thoracic CT patterns					
Pulmonary consolidations	6 (50)	3 (38)	4 (67)	3 (50)	0.79
Nodules and micronodules	6 (50)	2 (25)	2 (33)	2 (33)	0.79
Excavated pulmonary lesion	2 (17)	0	0	1 (17)	0.57
Ground glass opacities	4 (33)	3 (38)	0	2 (33)	0.40
Pleural effusion	1 (8)	2 (25)	1 (17)	2 (33)	0.67
Coinfections					
Bacterial	6 (38)	10 (63)	6 (55)	10 (83)	0.11
Viral	5 (31)	7 (44)	4 (36)	3 (25)	0.80
Fungal	7 (44)	7 (44)	5 (45)	8 (67)	0.61

Features	Hematologic malignancies, n = 16	Allo-HSCT, n = 16	Solid organ transplant, n = 11	Other, n = 12	p value
Acute kidney injury	7 (44)	11 (69)	11 (100)	11 (92)	**0.003**
Acute liver failure	6 (38)	5 (31)	6 (55)	1 (8)	0.11

*Data are no. (%). Bold text indicates statistical significance. Allo-HSCT, allogeneic hematopoietic stem cell transplantation; CT, computed tomography image.

Appendix Table 3. Coinfections in invasive fusariosis patients

Coinfections	No. patients (%), n = 55
Bacterial species	
Pseudomonas aeruginosa	9 (16)
Enterobacter cloacae	3 (5)
Enterococcus faecium	3 (5)
Escherichia coli	3 (5)
Klebsiella pneumoniae	3 (5)
Stenotrophomonas maltophila	3 (5)
Clostridium difficile	2 (4)
Enterococcus faecalis	2 (4)
Staphylococcus haemolyticus	2 (4)
Acinetobacter baumanii	1 (2)
Aeromonas hydrophila	1 (2)
Branhamella catarrhalis	1 (2)
Citrobacter koserii	1 (2)
Enterobacter aerogenes	1 (2)
Haemophilus influenzae	1 (2)
Klebsiella oxytoca	1 (2)
Pseudomonas alcaligenes	1 (2)
Staphylococcus aureus	1 (2)
Staphylococcus epidermidis	1 (2)
Streptococcus agalactiae	1 (2)
Streptococcus pneumoniae	1 (2)
Viral species	
Cytomegalovirus	12 (22)
Human herpesvirus 6	2 (4)
Coronavirus 229E	1 (2)
Herpes simplex virus	1 (2)
Human herpesvirus 8	1 (2)
Parainfluenza virus 2	1 (2)
Parainfluenza virus 3	1 (2)
Picornavirus	1 (2)
Respiratory syncytial virus	1 (2)
SARS-CoV2	1 (2)
Varicella zoster virus	1 (2)
Fungal species	
Aspergillus fumigatus	4 (7)
Absidia spp.	2 (4)
Candida albicans	2 (4)
Candida glabrata	2 (4)
Pneumocystis jirovecii	2 (4)
Rhizospus spp.	2 (4)
Alternaria spp.	1 (2)
Candida krusei	1 (2)
Candida parapsilosis	1 (2)
Candida lusitania	1 (2)
Scedosporium spp.	1 (2)

Appendix Table 4. Comparison (univariate analysis) of clinical features and therapeutic strategies between patients with clinical progression and patients with partial or complete response to therapy*

Characteristics	Progression, n = 28	Partial or complete response, n = 14	p value
Type of immunodeficiency			0.23†
Hematologic malignancy	6 (21)	6 (43)	
Recent allo-HSCT	12 (43)	2 (14)	
Solid organ transplant	5 (18)	2 (14)	
Other patients	5 (18)	4 (29)	
History of allo-HSCT‡	13 (46)	2 (14)	**0.049**
Diabetes mellitus	4 (14)	3 (21)	0.67
Immunosuppressive agents			
Corticosteroids >3 weeks	9 (32)	0 (0)	**0.019**
Other immunosuppressive therapy	14 (52)	3 (21)	0.09
Chemotherapy <3 mo	12 (43)	7 (50)	0.75
Biologic data			
Neutropenia, neutrophil count <0.5 g/L	14 (50)	4 (29)	0.32
Lymphopenia, lymphocyte count <1 g/L	27 (100)	10 (83)	0.09
Hypoalbuminemia, <35 g/L	26 (93)	11 (79)	0.31
Performance status at admission			**0.022**
>2	18 (64)	8 (57)	
<u><</u>2	10 (36)	6 (43)	
Median prognostic scores at admission (IQR)			
SAPS II	62 (49.5–69.5)	50 (37.5–57)	0.056
SOFA at admission	11.5 (9–16)	5.5 (3.5–7.5)	**0.002**
Clinical presentation			
Disseminated infection	10 (36)	1 (7)	0.07
Skin lesions	9 (32)	3 (21)	0.72
Pneumonia	21 (75)	11 (79)	1
Other	2 (7)	3 (21)	0.31
Thoracic CT patterns of fusariosis-related pneumonia			
Pulmonary consolidations	11 (73)	0	**0.001**
Nodules and micronodules	2 (13)	7 (88)	**0.001**
Excavated pulmonary lesions	1 (7)	1 (13)	1
Ground glass opacities	5 (33)	2 (25)	1
Pleural effusion	5 (33)	0	0.12
Antifungal treatment			0.27†
Monotherapy	19 (68)	9 (64)	
Combination	5 (18)	5 (36)	
None	4 (14)	0	
Type of molecule			
Voriconazole	14 (50)	11 (79)	0.09
Amphotericin B	16 (57)	9 (64)	0.74
Other	3 (11)	0	0.25

*Data are no. (%) except where indicated. Bold text indicates statistical significance. Allo-HSCT, allogeneic hematopoietic stem cell transplant; CT, computed tomography imaging; SAPS, simplified acute physiology score; SOFA, sequential organ failure assessment.
†From Fisher exact test; categories are mutually exclusive.
‡History of recent (<1 y) and past (>1 y) allogeneic stem cell transplant.

Appendix Table 5. Baseline characteristics and outcomes of patients according to ICU survival (univariate analysis)*

Characteristics	Died, n = 31	Survived, n = 24	p value
Median age, (IQR)	61 (53–70)	60 (51–66)	0.48
Sex			
F	7 (23)	9 (37)	
M	24 (77)	15 (63)	0.25
Type of immunodeficiency			0.12†
Hematologic malignancy	7 (23)	9 (38)	
Allo-HSCT	13 (42)	3 (13)	
Solid organ transplant	6 (19)	5 (21)	
Other patients	5 (16)	7 (29)	
Hematologic malignancies and allo-HSCT	20 (65)	12 (51)	**0.017**
Diabetes mellitus	5 (16)	5 (21)	0.73
Immunosuppressive agents			
Corticosteroids >3 weeks	9 (29)	3 (13)	0.19
Other immunosuppressive therapy	16 (53)	6 (25)	**0.05**
Chemotherapy <3 mo	13 (42)	11 (46)	0.79
Biologic data			
Neutropenia, neutrophil count <0.5 g/L	16 (52)	6 (25)	**0.047**
Lymphopenia, lymphocyte count <1 g/L	31 (100)	16 (89)	0.13
Hypoalbuminemia, <35 g/L	30 (97)	18 (78)	0.073

Characteristics	Died, n = 31	Survived, n = 24	p value
Antifungal prophylaxis	10 (32)	3 (13)	0.12
Performance status			0.08
>2	20 (65)	10 (42)	
<u><</u>2	11 (25)	14 (59)	
Median prognostic scores at admission (IQR)			
SAPS II	62 (49.5–68)	45 (38–53)	**0.007**
SOFA at admission	10.5 (8–15.5)	6 (4–9)	**0.001**
Ventilation			
Mechanical ventilation‡	26 (84)	18 (75)	0.50
Non-invasive ventilation	10 (32)	8 (33)	0.93
High flow nasal oxygen therapy	26 (84)	12 (50)	0.74
Prone position	3 (10)	2 (8)	1
Curare therapy	9 (29)	4 (17)	0.29
Nasal oxygenotherapy	27 (87)	22 (92)	0.69
Vasopressors	26 (84)	12 (50)	**0.006**
Acute kidney injury	25 (81)	15 (63)	0.14
Renal-replacement therapy	17 (55)	12 (50)	0.73
Acute liver failure	13 (42)	5 (21)	0.10
Time of diagnosis from ICU admission			0.93†
Before admission	6 (11)	6 (11)	
Day of admission	7 (13)	5 (9)	
After admission	17 (32)	12 (23)	
Clinical presentation			
Disseminated infection	9 (29)	3 (12.5)	0.19
Skin lesions	7 (23)	7 (29)	0.76
Pneumonia	25 (81)	17 (71)	0.52
Other	2 (6)	5 (21)	0.22
Thoracic CT patterns of fusariosis-related pneumonia			
Pulmonary consolidations	10 (56)	6 (43)	0.72
Nodules and micronodules	5 (28)	7 (50)	0.28
Excavated pulmonary lesions	0 (0)	3 (21)	0.08
Ground glass opacities	6 (33)	3 (21)	0.73
Pleural effusion	5 (28)	1 (7)	0.20
Bacterial coinfection	19 (61)	13 (54)	0.78
Viral coinfection	12 (39)	7 (29)	0.57
Fungal coinfection	15 (48)	12 (50)	0.91
Antifungal treatment			0.25†
Monotherapy	20 (25)	18 (78)	
Combination	7 (23)	5 (22)	
None	4 (13)	0 (0)	
Type of molecule			
Voriconazole	17 (55)	17 (71)	0.23
Amphotericin B	19 (61)	14 (58)	0.83
Other	3 (10)	0 (0)	0.25
G-CSF	8 (26)	4 (17)	0.47
Surgical debridement	0 (0)	7 (30)	**0.014**
Response to therapy			**<0.001**
Progression	25 (89)	3 (21)	
Partial or complete	3 (11)	11 (79)	

*Data are no. (%) except where indicated. Bold text indicates statistical significance. Allo-HSCT, allogeneic hematopoietic stem cell transplant; CSF, granulocyte colony-stimulating factor; CT, computed tomography imaging; ICU, intensive care unit; SAPS, simplified acute physiology score; SOFA, sequential organ failure assessment.
†From Fisher exact test; categories are mutually exclusive.
